# Cognate DNA Recognition by Engrailed Homeodomain Involves A Conformational Change Controlled via An Electrostatic-Spring-Loaded Latch

**DOI:** 10.3390/ijms23052412

**Published:** 2022-02-22

**Authors:** Nicola D’Amelio, Benjamin Tanielian, Mourad Sadqi, Pilar López-Navajas, Victor Muñoz

**Affiliations:** 1 IMDEA Nanoscience, Calle Faraday 9, Cantoblanco, 28049 Madrid, Spain; nicola.damelio@u-picardie.fr (N.D.); pilar.lopez.navajas@cib.csic.es (P.L.-N.); 2 Unité de Génie Enzymatique et Cellulaire UMR 7025 CNRS, Université de Picardie Jules Verne, 33 rue Saint-Leu, 80039 Amiens, France; 3 Chemistry and Biochemistry Graduate Group, University of California Merced, 5200 North Lake Road, Merced, CA 95343, USA; btanielian@ucmerced.edu; 4 CREST Center for Cellular and Biomolecular Machines, University of California Merced, 5200 North Lake Road, Merced, CA 95343, USA; msadqi@ucmerced.edu; 5 Department of Bioengineering, University of California Merced, 5200 North Lake Road, Merced, CA 95343, USA

**Keywords:** protein–DNA interactions, DNA recognition, transcription factors, DNA binding domains, conformational change, nuclear magnetic resonance, circular dichroism, control of gene expression, homeodomains

## Abstract

Transcription factors must scan genomic DNA, recognize the cognate sequence of their control element(s), and bind tightly to them. The DNA recognition process is primarily carried out by their DNA binding domains (DBD), which interact with the cognate site with high affinity and more weakly with any other DNA sequence. DBDs are generally thought to bind to their cognate DNA without changing conformation (lock-and-key). Here, we used nuclear magnetic resonance and circular dichroism to investigate the interplay between DNA recognition and DBD conformation in the engrailed homeodomain (enHD), as a model case for the homeodomain family of eukaryotic DBDs. We found that the conformational ensemble of enHD is rather flexible and becomes gradually more disordered as ionic strength decreases following a Debye–Hückel’s dependence. Our analysis indicates that enHD’s response to ionic strength is mediated by a built-in electrostatic spring-loaded latch that operates as a conformational transducer. We also found that, at moderate ionic strengths, enHD changes conformation upon binding to cognate DNA. This change is of larger amplitude and somewhat orthogonal to the response to ionic strength. As a consequence, very high ionic strengths (e.g., 700 mM) block the electrostatic-spring-loaded latch and binding to cognate DNA becomes lock-and-key. However, the interplay between enHD conformation and cognate DNA binding is robust across a range of ionic strengths (i.e., 45 to 300 mM) that covers the physiologically-relevant conditions. Therefore, our results demonstrate the presence of a mechanism for the conformational control of cognate DNA recognition on a eukaryotic DBD. This mechanism can function as a signal transducer that locks the DBD in place upon encountering the cognate site during active DNA scanning. The electrostatic-spring-loaded latch of enHD can also enable the fine control of DNA recognition in response to transient changes in local ionic strength induced by variate physiological processes.

## 1. Introduction

Transcription factors control gene expression by binding to specific DNA control elements and thereby recruit or block the recruitment of the transcription machinery to the target gene [1]. The DNA recognition process is carried out by the transcription factor’s DNA binding domain (DBD), which binds specifically to the cognate sequence with high (nanomolar) affinity [2]. DBDs also bind non-specifically to any other DNA sequence with much lower affinity, which results in sliding along DNA, a phenomenon thought to facilitate the stochastic search for the control element within the full genome [3]. The process of 1D diffusion along DNA has been thoroughly studied theoretically [4,5], using molecular simulations [6,7], and also experimentally via biochemical approaches [8], nuclear magnetic resonance [9], and single molecule fluorescence microscopy in vitro [10] and in vivo [11]. The binary interplay between cognate binding to a unique target and non-specific scanning has been found sufficient to explain the DNA recognition process of prokaryotic transcription factors in vivo [12].

Eukaryotic transcription is much more complex and involves control at multiple levels, including chromatic remodeling [13] and DNA methylation [14]. In addition, there are two major unsolved molecular puzzles about how eukaryotic DBDs effectively recognize their target sites. For instance, eukaryotic DBDs typically recognize short cognate sequences of less than 10 bp [15], which necessarily impairs specificity given that eukaryotic genomes contain tens of thousands of random occurrences of such short cognate sequences [16]. Furthermore, it has been recently discovered that eukaryotic DBDs bind DNA promiscuously rather than in binary fashion, and thus, exhibit a ladder of affinities ranging from specific to the cognate sequence (strongest) to completely non-specific (weakest) [17]. As a consequence, the DNA binding landscapes for these DBDs are highly rugged [17], and hence, much more difficult to navigate than anticipated. These observations suggest that eukaryotic DBDs may need a more sophisticated mechanism to effectively recognize their target sequences during DNA scanning.

The other intriguing puzzle refers to the potential role of conformational changes in the DNA recognition process. Protein conformational/allosteric control is thought to play multiple roles in eukaryotic transcription [18,19]. For DNA scanning, a possible such role involves intrinsically disordered regions adjacent to the DBD that scan the flanking DNA sequence via weak non-specific interactions, thus acting as monkey bars [20,21,22]. The monkey bar mechanism has been experimentally reported on oligomeric proteins such as p53 [23] and the lac repressor [24], or on tandem repeats of zinc-fingers [25]. The DBD, on the other hand, is assumed to be structurally rigid and to bind DNA canonically in a lock-and-key fashion. Such assumption is supported by structural studies showing DBDs folded into the same defined structure, whether alone [26] or bound to DNA [27,28]. However, there are several indications that eukaryotic DBDs are conformationally pliable. These domains share some sequence patterns with intrinsically disordered proteins, including low hydrophobicity and high net charge [29]. Calorimetric studies have reported partial disorder at physiological temperatures in eukaryotic DBDs [30,31]. Furthermore, nuclear magnetic resonance (NMR) structural studies of eukaryotic DBDs in absence of DNA have shown that, at the minimal ionic strength used in such NMR analyses, these proteins are marginally stable, or even unstable at room temperature [32,33,34].

**Figure 1:**
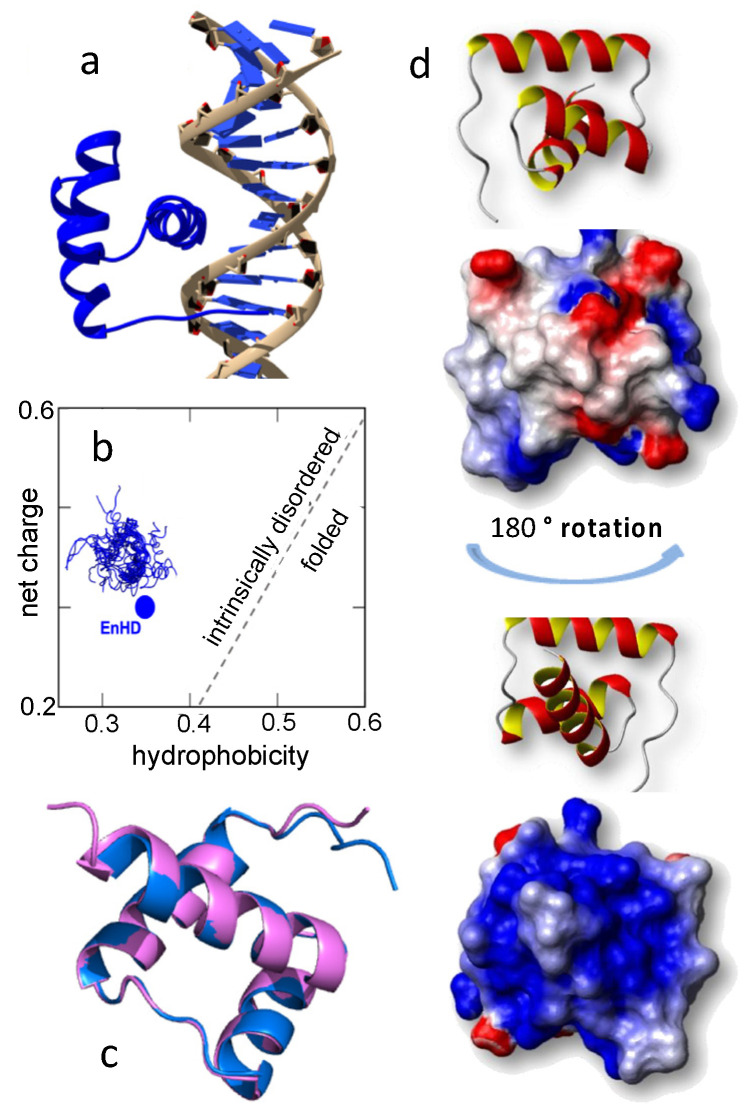
(**a**) X-ray structure of enHD bound to specific DNA (pdb code 1HDD) showing its three-helix bundle structure and binding via the insertion of the third α-helix into the DNA major groove. (**b**) EnHD amino acid sequence has the net charge versus hydrophobicity profile of an intrinsically disordered protein according to [29]. (**c**) Superposition of the X-ray structures of enHD bound to DNA (pdb code 1HDD, pink) and free (pdb code 1ENH, blue), both obtained at very high salt concentration. (**d**) Electrostatic potential of enHD in two orientations. The highly positively charged face that is involved in the interaction with DNA is displayed at the **bottom**.

Here we address these two puzzles by studying the interplay between the conformation of the DBD and its specific binding to the cognate DNA site. We focus on homeodomains, and particularly on the engrailed homeodomain (enHD), as a model for eukaryotic DBD. Homeodomains fold into an antiparallel three helix bundle that defines one of the major classes of DNA binding motifs in eukaryotic transcription [35]. They are typically found in transcription factors that operate as master regulators and are key players in embryonic development and morphogenesis [36]. Engrailed, in particular, controls over 200 genes in *Drosophila* [37] and defines embryonic parasegmental subdivision [38]. In humans, engrailed homeobox is linked to multiple defects in brain and eye development [39], as well as to many forms of cancer [40,41,42]. Biophysically, enHD epitomizes all of the special properties mentioned above for eukaryotic DBDs. It recognizes a very short (6 bp) palindromic cognate sequence (TAATTA) [43] and binds to specific DNA by superficially inserting its C-terminal helix into the DNA major groove (Figure 1a). The enHD sequence can be classified as intrinsically disordered on the basis of its net charge and hydrophobicity (Figure 1b). The X-ray structures of enHD in complex with DNA [28] and free [26], both of which were obtained at very high ionic strength [44], are nearly identical (Figure 1c). These 3D structures reveal a highly repulsive electrostatic potential due to the accumulation of positive charge on the face that interacts with DNA (Figure 1d), suggesting that the native structure of enHD is subjected to significant electrostatic strain. Consistently with this idea, protein engineering studies have shown that the enHD fold is marginally stable at room temperature [45], but can be significantly stabilized by reengineering its electrostatic potential via mutation [46].

To characterize the interplay between cognate DNA binding and conformation on enHD we employ NMR and circular dichroism. NMR is well suited for the structural analysis of the relatively small DBDs, including homeodomains. NMR has hence been widely used to structurally characterize DBDs and their interactions with DNA, starting with work in the early 90 s on the free DBDs (e.g., [32,47,48]), as well as subsequent studies of their complexes with DNA: see [49,50,51] as some examples. Such works have shown that DNA recognition often involves electrostatic interactions of the protein with the DNA phosphate backbone in which arginine and lysine side chains play a major role [52,53]. NMR has also been used to demonstrate that the lac repressor exhibits different conformational dynamics when bound specifically or non-specifically to DNA [54]. Finally, NMR relaxation experiments, particularly paramagnetic relaxation enhancement, can be utilized to resolve the 1D diffusive dynamics of homeodomains on non-specific DNA [55] as well as the translocation between cognate sites [53].

## 2. Results

### 2.1. The Conformational Ensemble of EnHD Is Modulated by Ionic Strength

**Figure 2:**
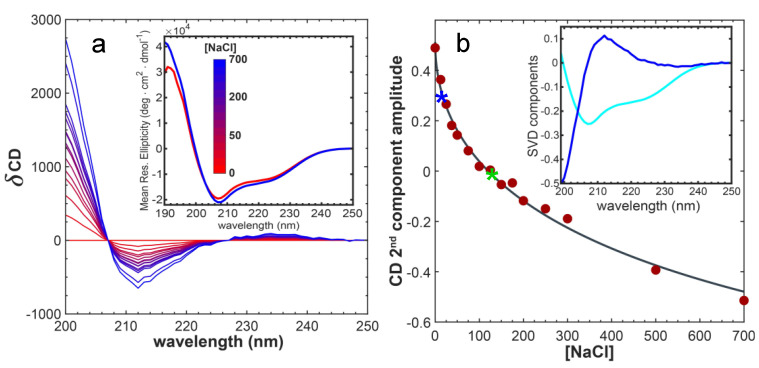
(**a**) Difference far-UV circular dichroism (CD) spectra of enHD as a function of NaCl concentration. The inset shows the spectra at the two extremes (0 and 700 mM NaCl). (**b**) Singular value decomposition of the CD spectra of enHD as a function of NaCl. The two first components are shown in the inset (first: cyan, second: blue) and the amplitude of the second component is given in the main panel. The black curve corresponds to a fit to the Debye–Hückel ionic strength equation. The two stars signal the salt concentrations of the NMR spectra in panel c (same color code).

The 3D structure of enHD reveals a highly positive electrostatic potential on the face that directly interacts with DNA (Figure 1d). To test the effect of electrostatic strain on enHD, we studied how its conformational ensemble is affected by changes in salt concentration (ionic strength) in absence of DNA. Far-UV circular dichroism (CD) experiments indicate that enHD has about 30% α-helical content at low ionic strength (inset to Figure 2a), which is lower than the helix content in the X-ray structure. We also observed that raising the ionic strength results in a slight monotonic increase in the α-helix signal (Figure 2a). Analysis of the CD spectra as a function of NaCl concentration by singular value decomposition reveals that the spectral change (blue in the inset of Figure 2b) is due to exciton effects indicative of increasingly rigid, or straighter, helices as salt concentration grows. This spectral change follows exactly the ionic strength dependence predicted by Debye–Hückel’s theory (Figure 2b), which demonstrates that it is triggered by ionic shielding of the electrostatic strain induced by the accumulation of positive charge on the 3D structure.

**Figure 3:**
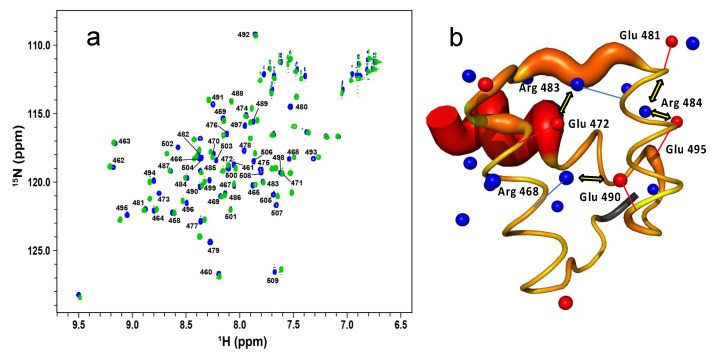
(**a**) ^1^H-^15^N HSQC spectrum of enHD at 100 μM concentration in 20 mM MES buffer at pH 6, and 25 mM NaCl (blue) or 120 mM NaCl (green). Labels indicate the assignments of enHD residues numbered according to the full engrailed sequence. (**b**) Changes in amide chemical shifts in response to ionic strength projected onto the X ray structure of free enHD (pdb code 1ENH). The thickness and color gradient (yellow to red) of the backbone reflects the combined ^1^H and ^15^N chemical shift deviations observed in the HSQC spectrum with increasing NaCl concentration in a log scale. The side chain nitrogen of arginine and lysine residues and sidechain oxygen of glutamate residues are displayed as blue and red spheres, respectively. The sidechain ion pairs that form salt bridges in the native enHD structure are indicated with double headed arrows, and their charged atom is connected to the corresponding Cα by a thin line of the same color for reference.

We then investigated the response to ionic-strength by NMR. Figure 3a shows the ^1^H-^15^N HSQC spectrum of enHD at pH 6 in the presence of 25 mM (blue) and 120 mM NaCl (green). We used pH 6 for NMR analysis because it was the value closest to physiological pH that reduced solvent exchange enough to resolve all the backbone amide peaks in the HSQC spectrum, even at low salt concentrations. The difference in ionic strength between these two experiments results in noticeable changes in the HSQC spectrum that are suggestive of a moderate increase in structure (more spectral spread) at higher ionic strength. These spectra correspond to conditions that amount to about one third of the total structural change observed by CD in the 0 to 700 mM range (stars in Figure 2b). Further consistency with CD results is provided by the observation that the HSQC spectrum of enHD continues to change as ionic strength increases further (secondary chemical shifts for 700 mM NaCl are discussed in Section 2.3). Mapping of these secondary chemical shifts onto the native structure reveals that most of the structural change concentrates on the regions with the most positive charge, including helix 3 (mainly is C-terminus), the end of helix 1 and the loop region connecting helices 1 and 2 (Figure 3b).

### 2.2. Binding to Cognate DNA Induces a Conformational Change on EnHD 

**Figure 4:**
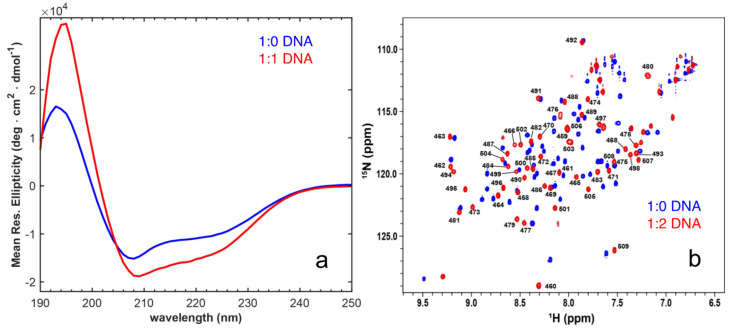
(**a**) Far-UV circular dichroism spectrum of enHD with 25 mM NaCl in the absence of DNA (blue) and in the presence of DNA at a 1:1 ratio (red). (**b**) ^1^H-^15^N HSQC spectrum of EnHD in 20 mM MES buffer at pH 6 and 25 mM NaCl, in the absence of DNA (blue) and in the presence of a two-fold excess of DNA (red). Numbered labels indicate the residue assignment in reference to the full engrailed sequence.

The X-ray crystallographic structures of enHD free and in complex with DNA (Figure 1c) are perfectly superimposable, indicating that the protein binds to DNA via a canonical lock-and-key process. However, when we studied binding to the exact same cognate DNA molecule used for the crystallographic studies, but in solution at physiological pH and low ionic strength (25 mM NaCl), we observed that enHD undergoes a large conformational change upon binding. We performed CD experiments at a protein concentration of 15 μM free and with DNA at a 1:1 ratio. The latter corresponds to saturating conditions for binding given that at this ionic strength the affinity of enHD for cognate DNA is nM [17,43]. These experiments were run with a spectral window from 300 to 190 nm, including a reference with only DNA (Supplementary Figure S1), and the spectral features of protein and DNA were separated by singular value decomposition of the combined basis set (see methods). The resulting far-UV CD spectra of enHD indicate that its conformation is strongly affected by the binding to DNA. Particularly, the spectrum of the protein in complex with DNA shows a strong enhancement of the α-helical signal, as judged by the intensity of the maximum at 194 nm and the minima at 208 and 222 nm (Figure 4a). This spectral change is consistent with an increase from 30% to ~50% in helix content. The trend is similar to that of the response to ionic strength (Figure 2a,b) but much more pronounced, indicating that the protein backbone becomes structurally more regular (longer/straighter α-helices) upon binding to DNA. 

**Figure 5:**
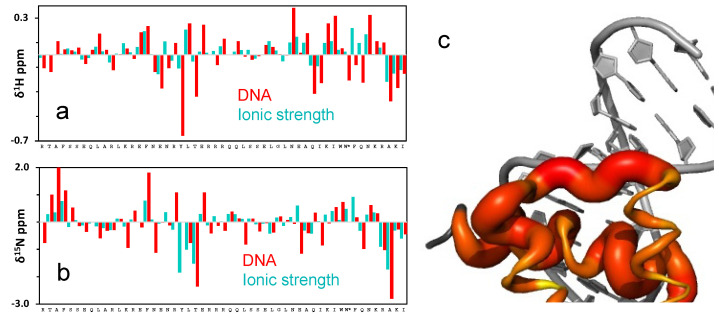
(**a**) Difference in the ^1^H chemical shifts of enHD in the presence of DNA (red, from panel (**a**)) or at 700 mM NaCl (cyan) relative to the spectrum of free enHD at 25 mM NaCl. The tryptophan sidechain chemical shift is also shown and indicated with a star. (**b**) As in (**a**) but for ^15^N chemical shifts. (**c**) Changes in amide chemical shifts upon binding to cognate DNA projected onto the X ray structure of free enHD (pdb code 1ENH). The thickness and color gradient (yellow to red) of the backbone reflects the combined ^1^H and ^15^N chemical shift deviations observed in the HSQC spectrum on a log scale.

The conformational change of enHD is also very noticeable by NMR, resulting in a ^1^H-^15^N-HSQC spectrum of the protein bound to DNA that is quite different from that obtained in absence of DNA (Figure 4b). Consistently with the strong binding expected at these conditions, the amide peaks in the presence of DNA are all well-defined without signs of line broadening. Overall, binding to DNA results in generalized changes of large magnitude in the backbone amide chemical shifts, with an average secondary shift of ~0.4 ppm for ^1^H (Figure 5a) and of ~1.3 ppm for ^15^N (Figure 5b). The largest secondary shifts are found again in residues located on the enHD regions that concentrate the net positive charge, including those most sensitive to ionic strength plus the N-terminus and the full helix 3 (Figure 5c). These regions lie on the face of the protein that is in direct contact with DNA in the X-ray structureand engage in specific interactions with the major groove through helix 3, or in electrostatic interactions with the DNA backbone. 

The DNA-bound spectrum is much more spread out (Figure 4b), which indicates a more anisotropic and/or structurally defined environment upon binding to DNA. The facts that the secondary shifts extend over the entire protein, including regions not in direct contact with DNA (Figure 5a,b), and that they also happen (albeit to a lesser extent) in the free protein in response to high ionic strength (Figure 3), suggest that the change in HSQC spectrum upon binding to cognate DNA primarily reflects a global conformational change of the protein. It is important to note that the direction of the changes in chemical shifts of the DNA-bound form and those induced by ionic strength in the free protein is sometimes aligned, but for some residues, the chemical shifts go in opposite directions (Figure 5a,b). Despite a commonality in the general trend, the changes with cognate DNA are much more extensive and marked (compare Figure 3b and Figure 5c). The direction reversal for some residues also highlights that there are key structural differences between the two conformational ensembles.

### 2.3. The Role of Electrostatics in EnHD’s Binding to Cognate DNA 

The electrostatic potential of enHD (Figure 1d) must play an important role in defining the DNA binding process. The X-ray structure of the complex shows the sidechains of R484 and R506 in close enough distance with the DNA phosphate backbone to establish salt bridges. There should also be an overall electrostatic stabilization of the complex arising from the mutual neutralization of charge between enHD and the DNA. To investigate the thermodynamic significance of these electrostatic effects by NMR we could not work at neutral pH. The reason is that the exchangeable protons of the positively charged lysine and arginine sidechains are usually not observable due to extensive exchange with the solvent. This phenomenon does happen for enHD both in absence of (Figure 3a) and bound to (Figure 4b), cognate DNA. However, at relatively low pH (4) the amide protons of the arginine residues in enHD exchange sufficiently slowly with the solvent as to be visible by NMR. Hence, we investigated the interactions between arginine sidechains and DNA by titrating enHD at pH 4 with cognate DNA. We performed these experiments in the presence of 700 mM NaCl so that we could study the interaction in conditions that more closely mimic the very high salt concentrations used in the X-ray structural analysis of enHD free and in complex with DNA [28,44].

**Figure 6:**
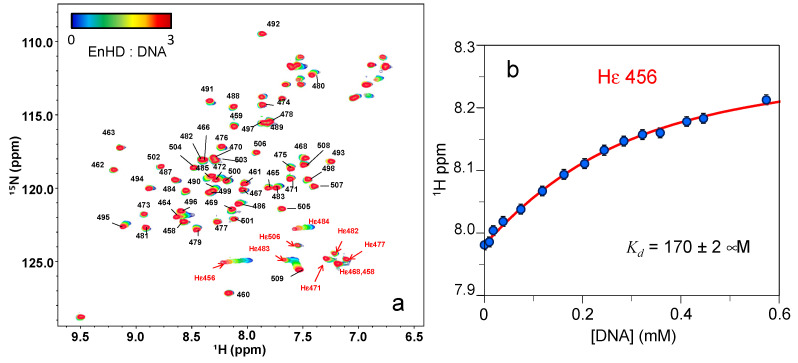
(**a**) Series of ^1^H-^15^N-HSQC NMR spectra of enHD (0.2 mM in 20 mM acetate buffer at pH 4) at high NaCl concentration (700 mM) and different concentrations of cognate DNA up to a 1:3 ratio of enHD to DNA. Numbered black labels indicate the residue assignment in reference to the full engrailed sequence. Red labels indicate the tentative assignments for the εNH of the nine arginine residues (456, 458, 468, 471, 477, 482, 483, 484, 506). The color bar on the **top left** indicates the particular enHD to DNA ratio for each spectrum. (**b**) ^1^H chemical shift of the εNH of R456 as a function of DNA concentration fitted to an equilibrium binding equilibrium (red line, Equations (1) and (2)).

The overlaid ^1^H-^15^N HSQC spectra of enHD at different DNA concentrations are shown in Figure 6a. These spectra provide several important pieces of information. The first one is that the cross-peaks for the sidechain amides of the nine arginine residues in enHD are clearly observable at all conditions, and could be assigned (red labels in Figure 6a). The second observation is that some of these amides experience large changes in chemical shifts during the titration, indicating that they indeed engage in direct interactions with DNA at high ionic strength (700 mM). Inspection of the X-ray structure of the enHD-DNA complex reveals that there are four arginine sidechains in close enough contact with the DNA to interact with it: R456, R458, R484 and R506. Of those, R456 and R484 experience large chemical shift changes in response to increasing amounts of DNA (Figure 6a). R458 seems to also experience secondary chemical shifts, but it partially overlaps with nearby cross-peaks that make further analysis difficult. In general, these results indicate that, at high salt concentrations, enHD binds to cognate DNA in solution, consistent with the X-ray structure of the complex. In contrast, the R506 amide sidechain, which has a well resolved cross-peak, does not shift upon the addition of DNA (Figure 6a). This is the case even though it is also at close range of the phosphate backbone in the complex structure. From a protein structural viewpoint, the main difference is that R506, which is inserted into the enHD core, has a low B-factor in the structure of the free protein and its environment experiences minimal structural reorganization on the bound complex. In contrast, the other three (particularly the N-terminal R456 and R458) and their surrounding residues have much higher B-factors in the free structure (suggestive of structural disorder) that decrease significantly in complex with DNA. This difference could explain the chemical shift insensitivity to DNA of the R506 amide sidechain. An alternative explanation is that at these conditions (solution, pH 4 and 700 mM NaCl) the complex is mostly stabilized by the electrostatic interactions of the N-terminus with the C-terminus being, on average, more distant from DNA.

To characterize the thermodynamics of DNA binding at these high salt conditions we analyzed the changes in chemical shift occurring during the DNA titration using a simple thermodynamic binding model. Particularly, we fitted the chemical shift change (Δ*δ*) as a function of the complex concentration to Equation (1), which describes the binding equilibrium for a system in fast exchange conditions [56].

(1) $$∆\delta =\delta_{HG}\left(\frac{\left[HG\right]}{{\left[H\right]}_{0}}\right)$$

where *H* and *G* refer to the Host (enHD) and Guest (DNA), *HG* to the complex, and the “0” subscript refers to the analytical concentration of enHD. In the case of a simple 1:1 complex, the concentration of the HG species in solution is given by [56,57]:

(2) $$\left[HG\right]=\frac{1}{2}\left({\left[G\right]}_{0}+{\left[H\right]}_{0}+\frac{1}{K_{a}}\right)-\frac{1}{2}\sqrt{{\left({\left[G\right]}_{0}+{\left[H\right]}_{0}+\frac{1}{K_{a}}\right)}^{2}-4{\left[H\right]}_{0}{\left[G\right]}_{0}}$$

where *K_a_* is the inverse of the dissociation constant *K_d_*. We fitted these equations to the ^1^H chemical shift data of R456, which experiences the largest shift with DNA (Figure 6a). The model reproduces the data well, thus supporting the assumption of 1:1 binding, and confirming that binding is indeed in the sub-mM range: *K_d_* of 170 ± 2 μM (Figure 6b). Hence, at these conditions, the affinity of enHD for cognate DNA is very low. This is expected because such high ionic strength should largely screen the electrostatic interactions between enHD and the DNA phosphate backbone. We can thus conclude, that electrostatic interactions play a primary role in driving the binding of enHD to cognate DNA, in agreement with previous studies [17,58]. In addition, we should be mindful that at pH 4 the nitrogenous bases of DNA start to titrate. Partial ionization of the bases could destabilize the double-stranded DNA structure [59] and hence contribute to further decreasing binding affinity in these conditions.

### 2.4. Electrostatic Shielding Blocks EnHD Conformation Leading to Lock-and-Key Binding to Cognate DNA

The experiments discussed above provide another important piece of information regarding the nature of the conformational change that enHD experiences upon binding to cognate DNA. As shown in Figure 4b, the chemical shifts of backbone amides change quite dramatically when enHD binds to cognate DNA at low salt concentrations. In contrast, the DNA titration at 700 mM NaCl shows the backbone amide peaks being insensitive to the cognate DNA, even with DNA in three-fold excess (Figure 6a). It is also noticeable that, compared to the spectra of the free and DNA bound forms at low salt concentrations, at 700 mM NaCl the backbone chemical shifts at all DNA ratios are somewhat halfway. The implication is that the electrostatic shielding induced by high ionic strength stabilizes a more rigid conformational ensemble on enHD that blocks the structural rearrangement that takes place upon binding cognate DNA at low salt concentrations, and hence binding becomes lock and key. This important result confirms that the large changes in the ^1^H-^15^N HSQC spectrum of enHD at low salt concentrations upon binding cognate DNA are mostly a consequence of the conformational change coupled to binding (also seen by CD, Figure 4a), rather than being simply caused by magnetic de-shielding from the proximity of the DNA in the complex. The results in Figure 6 also explain why the conformational change is not observed in the X-ray structures of free and bound enHD (Figure 1c), since these structures were obtained at 74% NH_4_-phosphate [44]. Moreover, these results confirm that the conformational response to ionic strength (Figure 2 and Figure 3) is tightly intertwined with the DNA recognition process. We interpret these combined results as suggestive of a built-in mechanism for cognate DNA recognition that involves a conformational change driven by the competition between intra- and inter-molecular electrostatic interactions. In this mechanism, the electrostatic strain caused by the accumulation of positive charge on the DNA interaction face (Figure 1d) operates as a spring that pushes to unfold the protein. In the absence of DNA, the tertiary interactions that hold the enHD fold together act as a latch that keeps the electrostatic spring compressed and under tension. When enHD approaches the cognate DNA, the formation of specific electrostatic interactions with the DNA helix pops the latch, and enHD undergoes a conformational change as it inserts its third helix into the major groove at the cognate site. In contrast, high ionic strength screens the electrostatic potential, which minimizes the strain on the folded structure (dampens the spring) and weakens the interactions with DNA, hence blocking the mechanism. The consequence is that at high ionic strength the protein becomes conformationally unresponsive to cognate DNA binding.

### 2.5. Interplay between EnHD Conformational Dynamics and Cognate DNA Binding 

**Figure 7:**
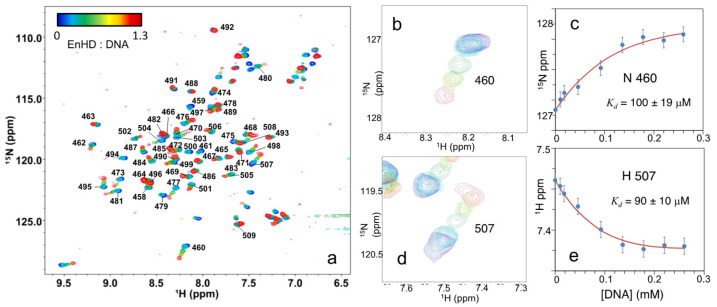
(**a**) Series of ^1^H-^15^N-HSQC NMR spectra of 3nHD (at 0.2 mM in 20 mM acetate, pH 4, NaCl 0.3 M and T = 298 K) at different concentrations of cognate DNA up to a 1:1.3 ratio of enHD to DNA. Numbered labels indicate the residue assignment in reference to the full Engrailed sequence. The color bar on the **top left** indicates the particular enHD to DNA ratio for each spectrum. (**b**) Detail of the spectral region corresponding to residue A460 (colors as in (**a**)). (**c**) ^15^N chemical shift of residue A460 as a function of cognate DNA concentration fitted to an equilibrium binding equilibrium (red line). (**d**) Detail of the spectral region corresponding to residue A507 (colors as in (**a**)). (**e**) ^1^H chemical shift of residue A507 as a function of cognate DNA concentration fitted to an equilibrium binding process (red line, Equations (1) and (2)).

If the electrostatic-spring-loaded latch mechanism outlined above is correct, one would expect the interplay between binding to cognate DNA and enHD conformation to be tunable by ionic strength. We hence performed a DNA binding titration of enHD at pH 4, to keep the arginine sidechain amides detectable, but at a significantly lower NaCl concentration (300 mM). The expectation would be that the lower ionic strength makes the binding stronger and the enHD conformational ensemble more flexible and responsive. The ^1^H-^15^N-HSQC spectra of enHD at different ratios of cognate DNA are shown overlaid in Figure 7a. This titration reveals significant changes relative to that at 700 mM. At these conditions, both the amides from arginine sidechains and backbone are sensitive to the addition of DNA. The peaks of sidechain amides for R456, R458, R484 and R506 (those in contact with DNA, see Section 2.3) are clearly visible in the absence of DNA, but experience loss of intensity and shifts in position upon addition of DNA (Figure 7a). These results suggest stronger binding in-between the fast and intermediate exchange regimes relative to the NMR timescale. More importantly, the backbone amides now become highly sensitive to DNA and move as the DNA ratio increases up to a 1:1 ratio, at which point the spectrum does not change anymore. This is consistent with the formation of a 1:1 complex like we concluded it happened at 700 mM NaCl. At intermediate DNA ratios, the peaks not only shift but also experience drops in intensity, confirming that this transition takes place at the interface between the fast and intermediate NMR exchange regimes.

At 300 mM NaCl, the ^1^H-^15^N-HSQC spectrum in the absence of DNA shows relatively small differences from that at higher salt concentrations (blue in Figure 5a and Figure 6a). On the other hand, the spectrum at saturating DNA conditions (red in Figure 5a and Figure 6a) differs significantly. It is in fact intermediate between the bound spectra at low ionic strength and pH 6 (Figure 4b) and that at 700 mM NaCl and pH 4 (Figure 6a). Such behavior is evident in the response of individual peaks, as can be seen for residues A460 and A507 in Figure 7b,d. These results confirm that high ionic strength rigidifies the enHD conformational ensemble, making it less responsive to cognate DNA binding. In other words, the presence of very high ionic strength changes the enHD conformational ensemble in a way that blocks its ability to rearrange during cognate DNA recognition. At this still high, but closer to physiological, ionic strength the enHD ensemble remains sufficiently flexible as to change conformation upon binding cognate DNA.

A460 and A507 are examples of residues with peaks that experience large chemical shift changes upon binding to DNA, but which can be resolved throughout the entire transition. The chemical shift curves for these residues confirm that the binding titration reaches a plateau at a 1:1 DNA ratio (Figure 7c,e), indicating saturating conditions from that point onward. We thus fitted these chemical shift curves to a 1:1 binding equilibrium using equations 1 and 2. The dissociation constant we obtained is in the micromolar range (95 ± 20 μM). This *K_d_* value is indeed consistent with NMR exchange conditions in between the intermediate and fast regimes: namely with cross-peaks that shift gradually upon addition of DNA but experience some line broadening (Figure 7c,e). It is also consistent with calculations with a statistical mechanical model of the affinity expected for enHD binding to cognate DNA at 300 mM NaCl [17]. Binding affinity at 300 mM NaCl is hence about 2-fold higher than at 700 mM, which agrees with the expected strengthening from simple Debye–Hückel screening.

### 2.6. Cognate DNA Recognition near Physiological Conditions

**Figure 8:**
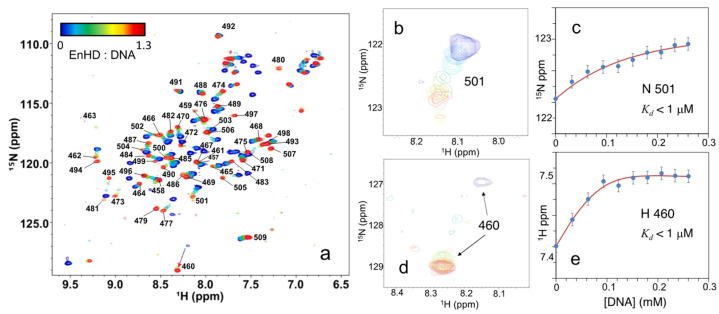
(**a**) Series of ^1^H-^15^N-HSQC NMR spectra of enHD (0.2 mM in 25 mM phosphate buffer at pH 6.8 and 50 mM NaCl) at different concentrations of cognate DNA up to a 1:1.3 ratio of enHD to DNA. Numbered black labels indicate the residue assignment in reference to the full engrailed sequence. The color bar on the **top left** indicates the particular enHD to DNA ratio for each spectrum. ((**b**) Detail of the spectral region corresponding to residue 501 (colors as in (**a**)). (**c**) ^15^N chemical shift of residue 501 as a function of cognate DNA concentration fitted to an equilibrium binding equilibrium (red line, Equations (1) and (2)). (**d**) Detail of the spectral region surrounding the A460 backbone amide cross-peak (colors as in (**a**)). (**e**) ^1^H chemical shift of A406 backbone amide as a function of DNA concentration fitted to an equilibrium binding equilibrium (red line, Equations (1) and (2)).

We then performed the same cognate DNA binding titration of enHD by NMR at even closer to physiological conditions by using 25 mM phosphate buffer at neutral pH (6.8) and 50 mM NaCl, which results in a net ionic strength of ~100 mM. At these conditions, the ^1^H-^15^N-HSQC NMR spectrum of enHD without DNA (blue in Figure 8a) is quite similar to that at pH 6 and low ionic strength (Figure 3a and Figure 4b). By the same token, the ^1^H-^15^N-HSQC NMR spectrum of enHD bound to DNA (red in Figure 8a) is very similar to that shown in Figure 4b for pH 6 and low ionic strength. The conformational changes occurring on enHD upon DNA binding are hence close to the maximal change defined by the low ionic strength conditions (e.g., Figure 4). At these nearly physiological conditions, the conformational ensemble of enHD is highly flexible and the affinity for the cognate DNA is sufficiently strong to pop the spring-loaded latch and trigger the conformational change of enHD upon binding. The strong thermodynamic coupling between binding and conformation results in drops in the intensity of many cross-peaks at the intermediate stages of the binding transition; particularly those that experience larger secondary shifts (Figure 8a). However, some of the backbone cross-peaks that experience large secondary shifts can be detected throughout the full transition, as for example residue W501 (Figure 8b). Others, such as A460, are barely visible in the absence of DNA, possibly due to a combination of solvent exchange in the free form and severe line broadening near the midpoint of the binding transition (Figure 8d). In general, these chemical shift patterns indicate that the DNA recognition process is taking place in the intermediate exchange relative to the NMR time scale, confirming binding with a much higher affinity.

We used the titration curves of W501 and A460 as a proxy of the overall DNA recognition process. Although these chemical shift curves are reasonably well fitted to the same 1:1 binding model and Equations (1) and (2) (Figure 8c,e), under these conditions of intermediate NMR exchange the affinity for DNA is too high to evaluate the *K_d_*. We can, however, estimate that the data shown in Figure 8 correspond to a thermodynamic binding process in which the overall *K_d_* is in the sub-μM range. This estimate is consistent with the nM affinity of enHD for a 75 bp dsDNA carrying one cognate site that has been recently determined by fluorescence correlation spectroscopy at similar experimental conditions [17], particularly considering that the much longer DNA sequence of that study is expected to also contribute to enhancing overall affinity.

### 2.7. Multilevel Interplay between DNA Interaction Energetics and EnHD Conformation

**Figure 9:**
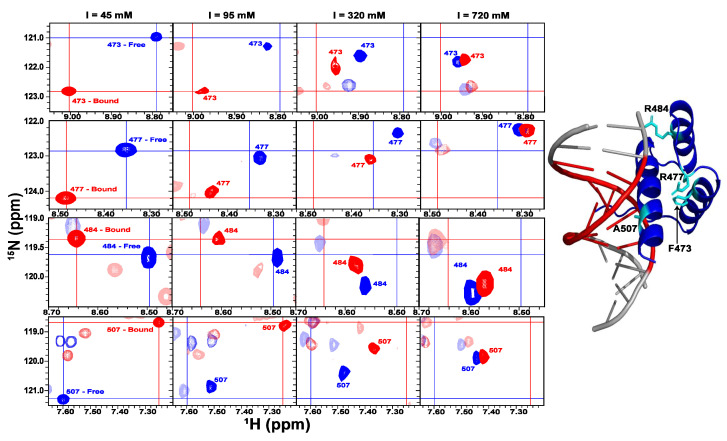
Interplay between ionic strength and cognate DNA binding in enHD monitored by NMR. (**Left**) each row shows the region of the ^1^H-^15^N-HSQC NMR spectra showcasing the amide cross-peak of one enHD residue at the four experimental conditions used in this study, corresponding to the ionic strengths indicated on **top**. Blue is for the free protein and red is for the protein in excess of cognate DNA. The crosses signal the center position at the lowest ionic strength for tracking purposes. The residues are (from **top** to **bottom**) F473, R477, R484, and A507. (**Right**) X-ray structure of the complex, which correlates with the highest ionic strength (rightmost) NMR spectrum, is shown with the 4 showcased residues in cyan.

Comparing the data at different experimental conditions reveals that the thermodynamic coupling between enHD conformation and the various energetic factors involved in cognate DNA recognition occurs at multiple levels. Figure 9 showcases this interplay through the NMR spectral changes of four exemplary residues. The four residues are located in regions of the protein that experience some of the largest spectral changes with ionic strength and cognate DNA binding. F473 is at the end of helix 1, R477 is in the loop connecting helices 1 and 2, R484 is at the beginning of helix 2. In none of these residues, the backbone is in contact with the DNA in the complex. A507 is located in helix 3 and inserts into the major DNA groove (Figure 9 right). To interpret the spectral changes structurally we use the highest ionic strength NMR spectra (rightmost column) as a reference equivalent to the conditions used in the determination of the X-ray structures of enHD free and in complex (i.e., Figure 1c).

The top row in Figure 9 shows the ^15^N and ^1^H chemical shifts of F473. In the free protein, the F473 peak moves towards random coil values as ionic strength drops, indicating the local unraveling of helix 1′s end. In the DNA-bound form the trend is the exact opposite, showing an increase in de-shielding that points to the extension of helix 1 into the loop. This interpretation is also consistent with the CD data for free and bound forms at low ionic strength. Therefore, electrostatic screening stabilizes a conformation around F473 that is approximately halfway of the change induced by specific binding to cognate DNA at low ionic strength. This residue is an example of symmetric effects on the enHD ensemble by both factors. 

The scenario is different for R477, which has chemical shifts close to random coil values at the high salt reference, and de-shields as ionic strength drops, both in the free and (much more markedly) in the bound forms. The simplest explanation for this result is that the loop connecting helices 1 and 2 unravels at low salt concentrations relative to the reference structure, possibly as a way to distance R477 from the positively charged side chains that surround it in the X-ray structure, and thereby reduce electrostatic repulsion. However, in doing so, the backbone of R477 becomes more solvent protected (e.g., hydrogen bonded). The much stronger de-shielding in the DNA-bound form suggests that the complex with DNA stabilizes helix 1 and extends it to fully incorporate R477, which is again consistent with the helical increase observed by CD at low salt concentrations (Figure 4a).

R488 exhibits yet another pattern in which ^15^N and ^1^H move with opposing trends. In the high salt structures, R484 is the first residue in helix 2 with carbonyl forming an i + 4 hydrogen bond. Its NH is also forming a hydrogen bond with the side chain of T481 in a typical helical N-cap motif. The ^1^H chemical shift moves with lower ionic strength similar to the pattern of F473: towards random coil in the free form, and towards more de-shielding in the bound form. On the other hand, the ^15^N chemical shift moves towards more structure (away from random coil values) in the free form, and even more markedly in the bound form. These combined changes suggest that at low salt concentrations without DNA, the helix 2 N-cap (which will affect mostly the ^1^H chemical shift) is broken, but the helical i + 4 hydrogen bond of its carbonyl remains formed (affecting primarily the ^15^N chemical shift). In contrast, binding to cognate DNA at low salt concentrations appears to extend helix 2 upstream so that the R483 backbone becomes double hydrogen bonded (carbonyl and NH) as expected for a central helical residue. Importantly, this interpretation also explains the changes in chemical shifts observed on T481, which experience the largest secondary shifts in the entire protein and move in the same direction for ionic strength and DNA binding (Figure 5a,b). Therefore, R483 showcases the competition between ionic strength and cognate DNA binding in stabilizing different conformational sub-ensembles in enHD: helix 2 N-capped at T481 versus a longer helix without N-capping (there are no suitable N-cap residues in the sequence upstream of T481).

A507 shows a trend similar to that of F473, namely ionic strength and cognate DNA move the ^1^H and ^15^N chemical shifts in the same direction. Indeed, at high salt concentrations, the A507 amide peak at both conditions nearly overlaps at a halfway position relative to the free and bound forms at low ionic strength. The changes in amide chemical shifts for this residue as a function of ionic strength and/or cognate DNA binding are among the largest in magnitude for the entire protein (Figure 5a,b), suggesting a large change in structure and/or local environment. The shift towards random coil values of the free form at low salt concentrations indicates that the end of helix 3 is mostly frayed at these conditions. In contrast, the structures at high salt concentrations show helix 3 extending all the way to the end of the enHD sequence. Moreover, A507 is at the interface of the interaction with the major groove, but it is too far away to engage in specific interactions (see Figure 9 right). Therefore, the enhanced shielding of the A507 amide when enHD is bound to DNA at low ionic strength possibly reflects ring current effects caused by a closer distance between A507 and the nitrogenous bases. This closer distance would imply a very different mode of interaction with cognate DNA; one in which helix 3 is slightly bent and more tightly wrapped around the major groove. Such type of interaction with cognate DNA would be more akin those found in the structures of the DNA complexes of several other helix-turn-helix motifs [60].

## 3. Discussion

Homeodomains are members of the helix-turn-helix family of DBDs typically found in eukaryotic transcription factors that function as master regulators [36]. Homeodomains are also relatively small domains (<60 residues) that are easily identified through sequence analysis, and which tend to be flanked by intrinsically disordered regions [61]. Accordingly, these domains have been center stage in the biophysical study of protein–DNA interactions; they were in fact among some of the first DBDs to be studied structurally, both by X-ray crystallography and NMR [61]. The picture that was painted from these structural studies is one in which the homeodomain folds into a defined three helix bundle structure and binds to cognate DNA by inserting its straight third helix into the DNA major groove in lock-and-key fashion (Figure 1a). The interactions that stabilize the complex combine electrostatic interactions of positively charged side chains with the phosphate backbone, and hydrogen bonds plus hydrophobic contacts between residues in the DBD and bases in the cognate site [60]. The first kind of interactions are sequence unspecific and provide the bulk binding affinity for DNA. The second kind provides additional affinity plus the specificity for the cognate sequence.

Non-specific binding is also important functionally because it allows the DBD to bind to any DNA sequence with low affinity and scan along its length by 1D diffusion, which is thought to accelerate the search for the target site [3]. It has been long recognized that the target search via 1D facilitated diffusion could benefit from a control mechanism on the protein that enables it to switch between non-specific DNA scanning and cognate binding modes, and hence lock into the target site upon encounter [62]. One example along these lines is the ordering-disordering transitions of the regions flanking the DBD, which for the lac repressor dimer have been found to be sensitive to whether the DBD is free, bound specifically, or bound non-specifically to DNA [54]. However, there is very little, if any, evidence of conformational changes taking place on the DBD itself upon binding. Here we have studied the interplay between cognate DNA binding and conformation of enHD. Our results provide direct biophysical evidence of a built-in mechanism by which this homeodomain changes conformation upon binding to cognate DNA. We also find that the conformational change is finely modulated by ionic strength, and operates as an electrostatic-spring-loaded latch that pops in response to the interactions formed between enHD and cognate DNA. In the following sections, we further discuss these findings, how they advance our understanding of the structural mechanisms for DNA recognition in eukaryotes, as well as comment on some thought provoking functional implications that emerge from these results.

### 3.1. A Conformational Ensemble with a Builtin Electrostatic-Spring-Loaded Latch

The amino acid sequence of enHD contains a total net charge of +7 at neutral pH (Figure 1b). A large fraction of that positive charge accumulates on the face that interacts with DNA, and is a key contributor to the DNA binding affinity, particularly through interactions between arginine side chains and the DNA phosphate backbone. The accumulation of net charge on a localized area of the protein surface is also likely to induce electrostatic strain on the structure. The strain should destabilize the folded state in the free form, which, together with the low intrinsic stability expected for homeodomains given their relatively small size [63], can result in an inherently pliable conformational ensemble. This expectation is consistent with protein folding studies that show that enHD’s native fold is marginally stable at neutral pH and 150 mM ionic strength: ΔG of 7.5 kJ/mol at 303 K or 3 *RT* [45]. Our results demonstrate that enHD does change conformation in response to ionic strength (Figure 2 and Figure 3). The change involves partial disordering at low ionic strength following a Debye–Hückel dependence, confirming that it is induced by ionic shielding of the electrostatic strain present in the folded structure. It is interesting to note that the conformational response to the ionic strength of enHD appears to be gradual rather than binary. The gradual nature of this conformational transition is apparent in the CD experiments (Figure 2b), and also in the chemical shift changes observed by NMR: e.g., compare the spectrum at 120 mM NaCl (Figure 3a) with the magnitude of the secondary shifts at 700 mM NaCl (Figure 5a,b). The gradual changes in chemical shift induced by ionic strength are also evident in Figure 9. Hence, the response of enHD to ionic strength appears to be consistent with a conformational rheostat rather than with a binary switch [64].

The overall change in enHD backbone conformation monitored by CD is of moderate amplitude relative to the total CD signal. Because the CD spectrum is averaged over all the peptide bonds in the protein, such modest amplitude could represent multiple scenarios. In one extreme, the observed change could indicate a slight reorganization of the entire backbone. On the other hand, it could point to a large structural transition focused on a small region of the protein. Our NMR data provide key additional information at the residue level that addresses this issue. The HSQC spectrum reveals that the conformational change in response to ionic strength is localized on specific regions of the protein, as shown in Figure 3b. The residues most involved are scattered throughout the sequence (N-terminus, loop connecting helices 1 and 2, and helix 3) but are structurally connected by their participation in defining the topology of the native fold (the orientation of the three helices) as well as the interaction interface with DNA. This region of enHD does accumulate most of the positive electrostatic potential present in the folded structure. In light of our results, we conclude that the energetic strain of bringing the three helices of enHD together via tertiary interactions operates as an electrostatic-spring-loaded latch that makes the native fold highly dynamic and primed to pop on cue. Further analysis of this region provides additional clues. Indeed, the available native structure of enHD (at very high salt concentrations) shows that the first two helices are connected by salt bridges between R468 and E490, and between E472 and R483; and that helix 3 interacts with helix 2 through another salt bridge between R484 and E495 (Figure 3b). We propose that this network of three long-range salt bridges is key to partially compensate for the overall electrostatic repulsion in this region and help define the overall orientation of the three helices (compress the spring). At low ionic strength, the electrostatic repulsion is strongest, and the enHD conformational ensemble is hence most heterogenous, flexible and dynamic. In these conditions, the three salt bridges (Figure 3b) can play an essential role in helping transiently populate the fold observed in structural studies at high salt concentrations. At high ionic strength, the screening of the electrostatic repulsive potential is maximal. However, the salt bridges should experience less screening because each salt bridge neutralizes the local net charge, and hence counterions will preferably accumulate around non-neutralized charges. In a high ionic strength scenario, the native tertiary interactions of enHD will then dominate, thereby stabilizing a structurally more rigid ensemble such as that of the X-ray structures (Figure 1c). This electrostatic-spring-loaded latch can make the conformational ensemble of enHD exquisitely sensitive to ionic strength, as we observe here.

### 3.2. Conformation versus Cognate DNA Recognition in EnHD

In this work we also demonstrate that enHD changes its conformation upon binding to cognate DNA. At first glance, these results appear in stark contrast with the essentially identical X-ray structures of enHD free and bound to DNA (Figure 1c). In fact, those X-ray structures played an important early role in establishing the idea that DBDs bind to cognate DNA as a key inserts in its lock. However, the crystallization conditions used for enHD include 74% NH_4_-phosphate [44], and hence an extremely high ionic strength. Our results highlight two implications for such crystallization conditions that had been unanticipated. First, high ionic strength makes the binding to cognate DNA extremely weak; for example, binding with hundreds of micromolar affinity at 700 mM (Figure 6). Second, the conformational ensemble of enHD is highly sensitive to ionic strength. We have shown that the combination of a weakened thermodynamic force for binding and rigidified enHD conformational ensemble does result in lock-and-key binding to cognate DNA in solution. Therefore, at equivalent conditions, the binding process in solution by NMR recapitulates the results of the earlier structural work.

However, as ionic strength decreases, resulting in proportionally stronger binding and a more flexible enHD ensemble, the thermodynamic coupling between these two processes becomes stronger. Their interplay demonstrates that enHD, and presumably other homeodomains, use conformational control in their DNA recognition process. Our results provide important structural and energetic clues of how such coupling arises from the complementarity in structure and interaction energetics between enHD and cognate DNA. The screening of the repulsive electrostatic potential by free counterions is by definition isotropic, weak, and cumulative. In contrast, an extended and mostly rigid polyanionic DNA neutralizes the positive charge of enHD through the concerted action of multiple anions organized around the spiraled backbone of the B-DNA helix. The electrostatic component of binding to DNA is, therefore, inherently cooperative, anisotropic, and expectedly stronger than simple Debye–Hückel screening. Furthermore, if the DNA happens to contain the cognate sequence (or to a lesser degree a partial consensus repeat, [17]), specific interactions with bases provide additional anisotropic stabilization. For instance, many of these specific interactions form between cognate bases and residues I500, W501, Q503, and N504 when the helix 3 of enHD inserts into the major groove. Other specific interactions involve the N-terminal R456 and R458 sidechains hydrogen bonding through the minor groove with two consecutive thymines placed on opposite strands, which provides further specific affinity for AT (or TA) repeats [27]. In conditions that make enHD flexible and binding strong (i.e., physiological ionic strength), these energetic factors cooperate to stabilize particular conformational sub-ensembles thereby optimizing the overall interaction potential. The interaction free energy is then sufficient to pop the spring-loaded latch so that enHD’s ensemble morphs in response to cognate DNA binding. The consequence of this sophisticated thermodynamic interplay is a powerful mechanism of conformational control of the DNA recognition process.

### 3.3. Functional Implications for an Interplay between EnHD Conformation and Interaction Energetics

Our analysis of the response of enHD to ionic strength and cognate DNA binding demonstrates the existence of a multilevel interplay between energetic factors that either compete or cooperate in modifying the enHD conformational ensemble. This multilevel interplay allows the homeodomain to respond/adapt to the local environment, a property they can use to scan DNA more efficiently and lock into target despite their very short recognition sequences. The functional implications are many and thought provoking.

The influence of the local environment on DNA recognition by transcription factors is an essential component of how they work but is a question that remains mostly unaddressed at the biophysical level. One important issue refers to what specific ionic strength(s) is (are) the most functionally relevant, and whether its local/transient changes affect the DNA recognition process. In cells, ion concentrations are tightly regulated by a complex system of pumps and channels. As a result, the average physiological ionic strength inside living cells is expected to be relatively stable at between 100 and 200 mM [65]. However, what is important for protein–DNA interactions is not so much the total ion concentration, but the ions that remain locally free in solution and those directly associated with the DNA region of interest. The free ions define the effective local ionic strength experienced by the free protein, whereas DNA-bound ions will compete with the protein for binding. Estimating these two ion fractions is not an easy task. Measurements of free ion concentrations using fluorescence biosensors estimate that the free ionic strength is somewhat lower [66]. For instance, the free ionic strength in the cellular cytoplasm is estimated to be around 110 mM [67]. Our results show that enHD exhibits a large conformational change upon binding cognate DNA at such ionic strengths (second column in Figure 9). Therefore, operation in standard physiological ionic strengths will afford enHD with robust conformational control of the DNA recognition process. On the other hand, the concentration of Na^+^ and K^+^ could be significantly higher in the cell nucleus, at least in the liver, kidney, thymus, and amphibian oocytes, resulting in overall nuclear ionic strengths of ~400 mM [68]. The conditions at the nucleus are obviously most significant for transcription factors, which lead us to nuclear free ionic strength of about 300 mM if we assume a similar 0.75 fraction of free ions. Importantly, our results show that, although toned down, the interplay between cognate DNA binding and conformation in enHD still occurs at 300 mM ionic strength (third column in Figure 9). Moreover, a free ion fraction of 0.75 is likely overestimated given the extremely high concentration of highly charged biomolecules (nucleic acids and histones) present in the nucleus, which will strongly adsorb free small ions.

The varying sensitivity of enHD’s conformation to ionic strength across the 40 to 300 mM range offers an intriguing mechanism for the fine control of DNA recognition through local/transient changes in ionic strength that is worth discussing further. For instance, even with the overall ionic strength being kept roughly constant, the availability of free ions is likely to experience large transient fluctuations at the local level in response to diverse physiological phenomena. EnHD could exploit its conformational-binding properties to modulate DNA recognition in fairly sophisticated and unanticipated ways in response to those local ion fluctuations. There are many functional instances in which one could envisage the utility of such a mechanism. For example, transcriptional activation of a given chromosomal region involves locally unpacking the chromatin and removing the nucleosomes to produce naked DNA [13]. This process will create a transient localized negative electrostatic potential along the naked DNA region and a delocalized positive potential around each of the dissociated histones. The histones will quickly draw freely diffusing small anions, depleting them from enHD molecules found in the vicinity. The local removal of counterions could then make enHD morph onto a “low ionic strength” ensemble and hence bind more tightly to the transcriptionally activated DNA region via a cooperative process. This and other conceivable functional modes are, of course, highly speculative at this point. However, what is certain is that the conformational-binding response of enHD that we report here provides the molecular mechanism to enable such functionalities.

Our results also shed important light on the mechanistic bases for efficient DNA scanning and target location by homeodomains. EnHD, and all other homeodomains, recognize very short (6 bp) cognate sites, which is puzzling given that eukaryotic genomes are very long and complex, but also repetitive. It has been recently discovered that the short cognate site makes enHD bind DNA promiscuously, that is, with a ladder of affinities that is proportional to the similarity to the cognate sequence [17]. That study also noted that highly promiscuous DNA binding enables the tracking of target genes because their regulatory regions contain long clusters of partial consensus repeats that operate as enHD attractors (i.e., DNA antennas) [17]. This is an exciting mechanism that potentially solves the gene tracking problem in eukaryotes. However, promiscuous DNA binding has an undesired kinetic byproduct: how to avoid getting stuck in the highly rugged binding landscapes resulting from the accumulation of partial consensus repeats in DNA antennas. In parallel, an earlier study using coarse-grained molecular simulations advanced a mechanism by which a gradually changing conformational ensemble (conformational rheostat) would allow enHD to dynamically adjust its scanning speed along non-specific DNA [69]. Interestingly, our results suggest how these two mechanisms can actually be connected. For instance, we found here that enHD experiences a large conformational change upon binding cognate DNA, provided that binding free energy is sufficiently strong to drive the change (i.e., moderate to low ionic strength). By the same token, weaker binding conditions (i.e., very high ionic strength) result in a different cognate binding mode in which enHD adopts the ensemble of the X-ray structures (Figure 1c) and interacts more superficially with DNA: mostly through electrostatic interactions of arginine sidechains (Figure 6). The latter binding mode could mimic how enHD binds to a fully non-consensus DNA sequence, which can only establish non-specific interactions and hence inevitably results in weaker binding. Likewise, binding to a partial consensus repeat will add some of the cognate-specific interactions, resulting in mid-level affinity. A binding free energy that varies with the DNA sequence can make enHD change conformation gradually, as it does when the cognate binding is weakened by increasing ionic strength (Figure 9). EnHD could exploit this behavior as a mechanism to scan DNA adaptively, reading out the sequence in real time by dynamically morphing its conformational ensemble in response to the detailed energetic balance provided by the interactions with DNA. This adaptive readout mechanism provides a simple strategy for efficient navigation of the rugged binding landscapes arising from promiscuous binding. The conformational change that takes place upon binding cognate DNA would then work as a signal transducer that locks enHD into the cognate site upon encounter, so it avoids bypassing the target. This hypothesis offers an exciting novel mechanism for the conformational control of DNA scanning in enHD. Such control is likely to be present in other homeodomains as well, given their structural and sequence commonalities [35]. This proposed mechanism needs to be further analyzed and confirmed in follow up biophysical and biological studies. However, it does have the fundamental advantage of effectively integrating all of the existing biophysical data on enHD folding and DNA binding, including the new data we present here. Furthermore, it can explain some of the puzzling characteristics of DNA recognition by transcription factors in eukaryotes.

## 4. Materials and Methods

### 4.1. Protein Expression and Purification

Codon-optimized gene sequences encoding for the 54-residue EnHD alone or as a fusion with the SUMO protein were synthesized and cloned into the pBAT vector (TopGene Tech, St-Laurent, Quebec, Canada). Both recombinant proteins (EnHD and SUMO-EnHD) were expressed in BL21(DE3) *E. coli* strain (Novagen, Gujarat, India). Bacteria were grown at 37 °C in minimal medium containing ^15^NH_4_Cl (Spectra Stable Isotopes, Columbia, MD, USA) as the sole nitrogen source or containing ^15^NH_4_Cl as the sole nitrogen source and ^13^C_6_-D-glucose (Spectra Stable Isotopes, Columbia, MD, USA) as sole carbon source. After reaching an OD600 of 0.8–1.0, protein expression was induced by adding 1 mM IPTG (isopropyl-β-D-thiogalacto-pyranoside). For sole EnHD, we continued expression after induction for 4 h at 30 °C to avoid protease degradation. The cells were harvested by centrifugation and resuspended in a 20 mM phosphate buffer at pH 7.0, containing 150 mM NaCl and 0.1 mM protease inhibitor cocktail (Sigma, St. Louis, MO, USA). Cells were then lysed by sonication at 4 °C and centrifuged at 25,000 rpm for 30 min. The supernatant was purified by cation exchange chromatography using an SP sepharose Fast Flow column (GE Healthcare, Chicago, IL, USA). A second purification step was necessary using reverse phase chromatography. The purified protein solution was lyophilized and was confirmed by electrospray mass spectrometry with >95% purity. For the SUMO-fused EnHD, we expressed the protein overnight at 37 °C given the higher stability of this construct. Cells were harvested by centrifugation and resuspended in B-PER Complete Bacterial Protein Extraction buffer (Fisher, Waltham, MA, USA). Cells were incubated in buffer for 60 min then centrifuged at 30,000 rpm for 30 min. The supernatant was purified using a HisTrap HP column (GE Healthcare, Chicago, IL, USA). The SUMO tag was cleaved by incubating the column’s elutant with ULP1 overnight at 4 °C. A second purification step using the HisTrap HP column was needed to remove the SUMO-tag. A third purification using a C4 reverse phase column (Higgins Analytical, Mountain View, CA, USA) was then performed to remove excess salt from the buffer. The sample was then lyophilized and confirmed by SDS-PAGE and electrospray ionization Mass Spectrometry with a purity >98%.

### 4.2. Cognate DNA Molecule

As a model of cognate DNA binding, we used the same DNA molecule of the earlier X-ray structural studies [27]. Particularly, the DNA sequence TTTTGCCATGTAATTACCTAA (from 5′ to 3′) and its complementary strand ATTAGGTAATTACATGGCAAA were purchased from either Sigma-Aldrich (St. Louis, MO, USA) or Integrated DNA Technologies (IDT). The double stranded DNA was hybridized by mixing equal volumes of 5 or 10 mM solutions of each oligonucleotide in the buffer used for the NMR experiments. DNA annealing was achieved by heating at 393 K for 10 min followed by slow cooling to sample down to 277 K using a T100 Thermal cycler from Bio-Rad (Hercules, CA, USA). Good stoichiometry and pairing were confirmed by analytical centrifugation.

### 4.3. Far UV Circular Dichroism (CD) Spectroscopy

Samples for the circular dichroism analysis of the ionic strength dependence were prepared at 40 µM EnHD in 20 mM Tris buffer at pH 7.5. The ionic strength was changed by adding different amounts of potassium fluoride (KF), which is the most UV-transparent of the monovalent salts, from a 700 mM stock solution prepared in the same buffer. Samples for the CD analysis of the effects of cognate DNA binding were prepared with only EnHD at 15 µM, as a negative control, cognate DNA at 15 µM (see below), for the subtraction of the inherent CD spectrum of DNA, and with both enHD and cognate DNA at equimolar concentrations. CD spectra were acquired on a Chirascan Plus spectrophotometer (Applied Photophysics, Leatherhead, UK). Data were collected with 1 nm resolution in the 190–250 nm range for the ionic strength dependence (2 s acquisition per nm), and in the 190–300 nm (10 s acquisition per nm) for DNA binding. The spectra were recorded using a 1 mm pathlength cuvette at room temperature, and buffer baseline was subtracted. The baseline subtracted data were transformed into mean residue ellipticity units, and then analyzed using singular value decomposition in MATLAB.

### 4.4. Nuclear Magnetic Resonance

NMR samples for resonance assignment were prepared with ^13^C, ^15^N uniformly labeled enHD in 20 mM acetate buffer, 0.1 mM NaCl, pH 4, 5% D_2_O/ 95% H_2_O. Under these conditions, enHD remains soluble and monomeric. NMR experiments were acquired at 294 K in a Bruker Avance III 600 MHz spectrometer equipped with a triple resonance triaxial-gradient probe. Sequence backbone chemical shift assignments were obtained from the experiments: [1H-15N]-HSQC, 3D HNCACB and 3D CBCA(CO)NH. NMR samples for cognate DNA binding experiments were prepared at a final concentration of 200 µM enHD in four different buffers: (i) 20 mM MES buffer at pH 6 plus 25 mM NaCl; (ii) 20 mM Acetate at pH 4 plus 0.3 M NaCl; (iii) 20 mM acetate at pH 4 plus 0.7 M NaCl; (iv) 25 mM phosphate at pH 6.8 plus 50 mM NaCl. All the NMR samples were prepared using a 5% D_2_O/95% H_2_O mixture. EnHD/DNA samples for each step in the binding titration were prepared by mixing solutions of enHD and hybridized cognate DNA at each suitable molar ratio but at concentrations 10-fold diluted relative to the target final concentration of 200 µM enHD. A volume of 3 mL of the 10× diluted sample was then concentrated down to 300 µL final sample volume using 3K MWCO Amicon Ultra Centrifugal Filters (Sigma, St. Louis, MO, USA) spun at 3000× *g* for 60 min at 4 °C. Sample preparation at 10-fold dilution avoids the precipitation of enHD during the mixing with DNA. [1H-15N]-HSQC spectra were acquired at 298K in a Bruker Avance III 600 MHz spectrometer. NMR data were processed using NMRPipe [70] and Sparky [71] was used to visualize the processed data.

## Supplementary Materials

The following supporting information can be downloaded at: https://www.mdpi.com/article/10.3390/ijms23052412/s1.
