# Supplementary material for: Cognate DNA Recognition by Engrailed Homeodomain Involves A Conformational Change Controlled via An Electrostatic-Spring-Loaded Latch
Source: Int J Mol Sci. 2022 Feb 22;23(5):2412. doi: 10.3390/ijms23052412 (PMC8910618; doi:10.3390/ijms23052412)
Supplement: Supplementary file 1 [file ijms-23-02412-s001.zip › ijms-1567538-supplementary.pdf]

Supplementary Information

Cognate DNA Recognition by Engrailed Homeodomain Involves a Conformational Change Controlled via an Electrostatic-Spring-Loaded Latch

Nicola D'Amelio, Benjamin Tanielian, Mourad Sadqi, Pilar López-Navajas, and Victor Muñoz

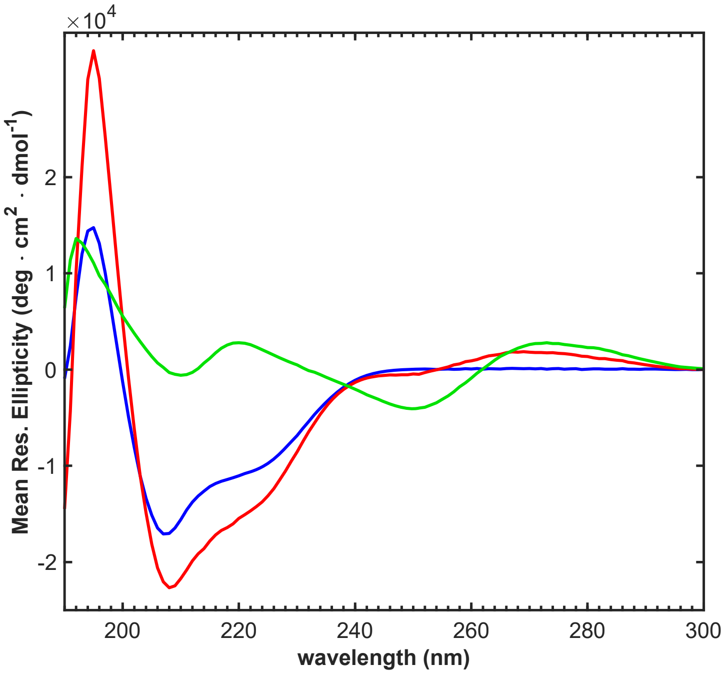

Figure S1: **Circular dichroism analysis of enHD and cognate DNA**. Near-far-UV circular dichroism spectra of samples with enHD and DNA in 20 mM MES buffer at pH 6, and 25 mM NaCl. (blue) enHD alone; (green) 21 bp cognate DNA alone; (red) mix of enHD and DNA at 1:1 ratio. All samples were prepared at 15 μM of protein and/or DNA.
